# The immune evasion protein Sbi of *Staphylococcus aureus* occurs both extracellularly and anchored to the cell envelope by binding lipoteichoic acid

**DOI:** 10.1111/j.1365-2958.2011.07966.x

**Published:** 2012-01-18

**Authors:** Emma Jane Smith, Rebecca M Corrigan, Tetje van der Sluis, Angelika Gründling, Pietro Speziale, Joan A Geoghegan, Timothy J Foster

**Affiliations:** 1 Microbiology Department, Moyne Institute of Preventive Medicine, Trinity CollegeDublin 2, Ireland; 2 Section of Microbiology, Imperial College LondonArmstrong Rd, London SW7 2AZ, UK; 3 Department of Molecular MedicineViale Taramelli 3/b, 27100 Pavia, Italy

## Abstract

The Sbi protein of *Staphylococcus aureus* comprises two IgG-binding domains similar to those of protein A and a region that triggers the activation of complement C3. Sbi is expressed on the cell surface but its C-terminal domain lacks motifs associated with wall or membrane anchoring of proteins in Gram-positive bacteria. Cell-associated Sbi fractionates with the cytoplasmic membrane and is not solubilized during protoplast formation. *S. aureus* expressing Sbi truncates of the C-terminal Y domain allowed identification of residues that are required for association of Sbi with the membrane. Recombinant Sbi bound to purified cytoplasmic membrane material *in vitro* and to purified lipoteichoic acid. This explains how Sbi partitions with the membrane in fractionation experiments yet is partially exposed on the cell surface. An LTA-defective mutant of *S. aureus* had reduced levels of Sbi in the cytoplasmic membrane.

## Introduction

*Staphylococcus aureus* permanently colonizes the moist squamous epithelium of the anterior nares of approximately 20% of the population while the remainder carry the organism intermittently (Williams, 1963; Peacock *et al*., 2001). Colonization is an established risk factor for development of infection both in the hospital and in the community (Lowy, 1998). *S. aureus* can cause a variety of infections ranging from superficial skin lesions such as boils and abscesses to invasive and potentially life-threatening infections such as osteomyelitis, septic arthritis and endocarditis (Petti and Fowler, 2003; Fowler *et al*., 2005).

The ability of *S. aureus* to cause infections is in part due to proteins that are anchored to the cell surface and to those that are secreted into the medium. Among the latter are cytolytic toxins, enzymes and proteins with immune evasion functions that interfere with neutrophil migration and complement fixation (Foster, 2005). While a major function of surface-anchored proteins is to act as adhesins and invasins (Foster, 2005), it is also clear that several can also help the bacterium evade innate immune responses. Thus protein A binds to the Fc region of IgG and coats the cell with antibody that cannot be recognized by Fc receptors on neutrophils and cannot catalyse complement fixation. Clumping factor A binds fibrinogen and fibrin (McDevitt *et al*., 1997) but it can also capture and activate the complement regulatory protease factor I which results in enhanced degradation of C3b (Cunnion *et al*., 2004; Hair *et al*., 2008).

Proteins can be anchored to the cell envelope of Gram-positive bacteria by several mechanisms (Cabanes *et al*., 2002). (i) Covalent linkage to cell wall peptidoglycan occurs by the action of sortase on the LPXTG motif that is part of a C-terminal wall-anchoring domain (Mazmanian *et al*., 2001). The sorting signal also comprises a hydrophobic membrane-spanning domain followed at the extreme C-terminus by positively charged residues. (ii) Lipoproteins are possibly anchored to the outer face of the cytoplasmic membrane (Inouye *et al*., 1977; Bubeck Wardenburg *et al*., 2006). The signal peptide is typically shorter than that of proteins that are secreted into the medium and is followed by a cysteine residue. Lipoprotein diacylglycerol transferase catalyses transfer of diacylglycerol from phosphatidylglycerol in the outer face of the membrane to the sulphydryl moiety of the cysteine followed by cleavage of the signal peptide by signal peptidase II (Tokunaga *et al*., 1982; Choi *et al*., 1986; Gan *et al*., 1993). (iii) Proteins may be anchored non-covalently to the cell wall components peptidoglycan and teichoic acids. Internalin B (InlB) of *Listeria monocytogenes* has C-terminal ‘GW’ repeat domains of ∼ 80 residues that bind to lipoteichoic acid (LTA) (Jonquieres *et al*., 1999). Thus InlB is associated with the cytoplasmic membrane in cell fractionation experiments but can also occur extracellularly (Braun *et al*., 1997). Furthermore, cell-bound InlB can be displaced by soluble LTA and by highly negatively charged heparin sulphate proteoglycan (Jonquieres *et al*., 1999). Autolysins such as Alt from *S. aureus* and AtlE from *Staphylococcus epidermidis* are also attached to the cell envelope via GW repeats (Oshida *et al*., 1995; Heilmann *et al*., 1997). (iv) The extracellular adherence protein Eap (also known as Map) has repeated domains that can bind to several different ligands (Chavakis *et al*., 2002). The protein can also bind to the bacterial cell surface by an unknown mechanism and promote attachment to and invasion of mammalian cells, most likely by binding to fibronectin and forming a bridge to the α5β1 integrin (Harraghy *et al*., 2003; Hussain *et al*., 2008).

**Fig. 1:**
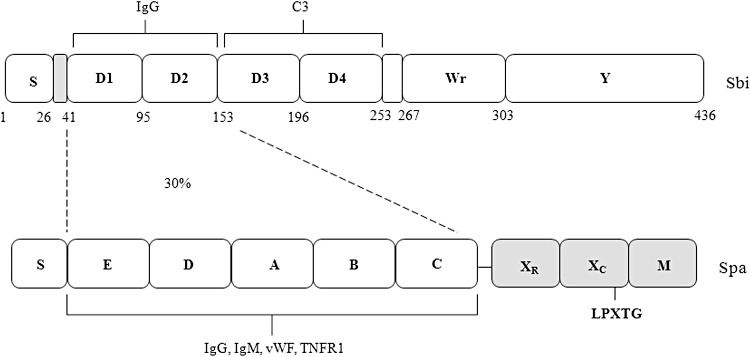
Schematic diagrams of Sbi and Spa. The upper figure is Sbi and the lower is Spa. S, signal sequence; D1D2, Sbi IgG-binding domains that have sequence similarity to the IgG-binding domains of Spa (E, D, A, B, C); D3D4, Sbi complement factor C3-binding domains; Wr and Xr, proline-rich C-terminal domains; Y, C-terminal domain; LPXTG, wall-anchoring motif; M, transmembrane domain and positively charged C-terminus. Spa ligands are indicated.

The second binding protein for immunoglobulins (Sbi) comprises four N-terminal ligand-binding repeats. Its C-terminus comprises the proline-rich region Wr and the tyrosine-rich region Y that is assumed to be involved in Sbi association with the cell envelope (Fig. 1; Zhang *et al*., 1998). The first two N-terminal repeats (D1 and D2) have sequence similarity to the IgG-binding domains of protein A. Indeed the predicted structures show that residues on the faces of helices I and II of Spa and Sbi are conserved, allowing Sbi to bind to IgG in a similar fashion to the archetypal LPXTG-anchored immune evasion protein. Domains D3 and D4 are also separately folded and contribute to the elongated structure of the protein (Burman *et al*., 2008; Upadhyay *et al*., 2008). They bind to complement protein C3. It has been argued that binding to and promoting the conversion of C3 to C3b would only be an effective immune evasion mechanism if the protein were extracellular (Burman *et al*., 2008).

Previously we reported a detailed and systematic analysis of the cellular location of Sbi (Smith *et al*., 2011). By analysing mutants lacking Sbi and protein A we were able to demonstrate that Sbi occurs both extracellularly and bound to the cell envelope and that both contribute to immune evasion. By expressing truncated Sbi in *S. aureus* and using purified recombinant Sbi we show that the C-terminal Y domain is required for attachment to the membrane. This is likely to be mediated by its interaction with lipoteichoic acid.

## Results

### Surface expression of Sbi D3D4 ligand-binding domains

**Fig. 2:**
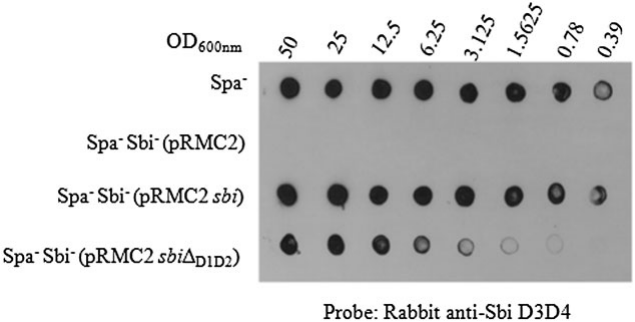
Surface expression of Sbi domains D3D4. Serial dilutions of cells were applied to a nitrocellulose membrane and probed with rabbit anti-Sbi D3D4WrY IgG followed by HRP-conjugated goat anti-rabbit IgG.

Previously we reported the surface exposure of the IgG binding D1D2 domains of Sbi (Smith *et al*., 2011). In order to determine if the C3-binding domains of Sbi were exposed on the cell surface whole cells were immobilized on membranes and probed with rabbit antibodies raised against the non-IgG binding D3D4 domains of Sbi. Cells expressing wild-type Sbi could bind antibodies via domains D1 and D2 by a non-immune reaction as well as by a specific immune reaction with domains D3 and D4. The same reactivity was seen with cells expressing wild-type Sbi from the chromosomal gene and when induced from pRMC2*sbi*^+^ (Fig. 2). In contrast cells expressing a mutant of pRMC2*sbi^+^* which expressed a truncate that lacked the IgG binding D1D2 domains reacted 16- to 32-fold less. Given that D1 and D2 can each bind to a single Fc region each whereas D3D4 most likely has several epitopes for polyclonal IgG Fab it is possible that the majority of D3D4 are buried within the cell wall and are not exposed on the cell surface.

### Sbi binding to the cytoplasmic membrane

**Fig. 3:**
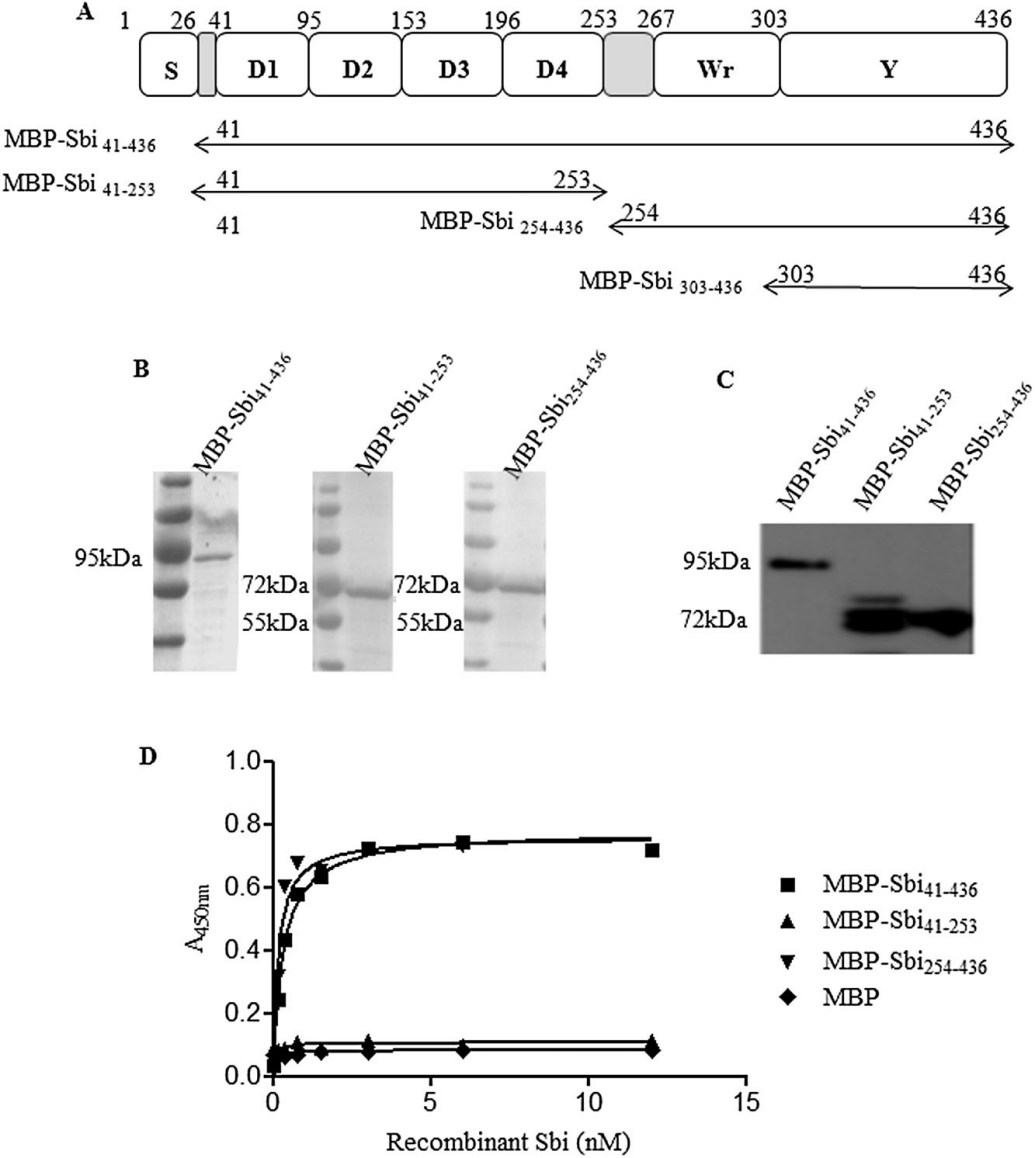
Binding of MBP–Sbi_41–436_, MBP–Sbi_41–253_ and MBP–Sbi_254–436_ to purified cytoplasmic membrane. A. Schematic diagram of Sbi showing the residues present in each recombinant MBP-tagged protein. B. Coomassie stain of an SDS-PAGE gel of MBP–Sbi recombinant proteins. C. Western immunoblot of MBP–Sbi recombinant proteins probed with HRP-conjugated anti-MBP IgG. D. Binding of MBP–Sbi_41–436_, MBP–Sbi_254–436_ MBP–Sbi_41–253_ and MBP to wells coated with cytoplasmic membrane material isolated from Newman Spa^-^ Sbi^-^ cells. Recombinant protein binding was detected with HRP-conjugated anti-MBP IgG. Binding assay was preformed *n* = 3 times with similar results. The graph shown is a representative of one experiment with each point plot representing the average of duplicate wells.

To address the importance of the C-terminal domain of Sbi in membrane anchoring, three maltose-binding protein (MBP) fusion proteins were constructed (Fig. 3A). These comprised the entire Sbi protein (residues 41–436), the N-terminal ligand-binding domains (residues 41–253) and the C-terminal domains Wr and Y (residues 253–436). The proteins were expressed in *Escherichia coli* and purified by affinity chromatography. Their purity and integrity were verified by SDS-PAGE (Fig. 3B) and Western blotting with anti-MBP antiserum (Fig. 3C).

Cytoplasmic membrane material purified from *S. aureus* Newman Spa^-^ Sbi^-^ was incubated in microtitre plates and coating of the surface was verified with antibodies recognizing the integral membrane protein EbpS (data not shown). The membranes were incubated with MBP–Sbi_41–436_ and MBP–Sbi_254–436_ which were able to bind in a dose-dependent and saturable manner with half maxima of 0.54 ± 0.1 nM and 0.57 ± 0.1 nM, respectively, while MBP–Sbi_41–253_ and the MBP control were unable to bind (Fig. 3D). These results indicate that the C-terminal WrY domain of Sbi binds to purified cytoplasmic membrane mimicking precisely the results seen with fractionated *S. aureus* cells expressing Sbi truncates.

### Recombinant Sbi binds to whole cells and fractionates with the cytoplasmic membrane

**Fig. 4:**
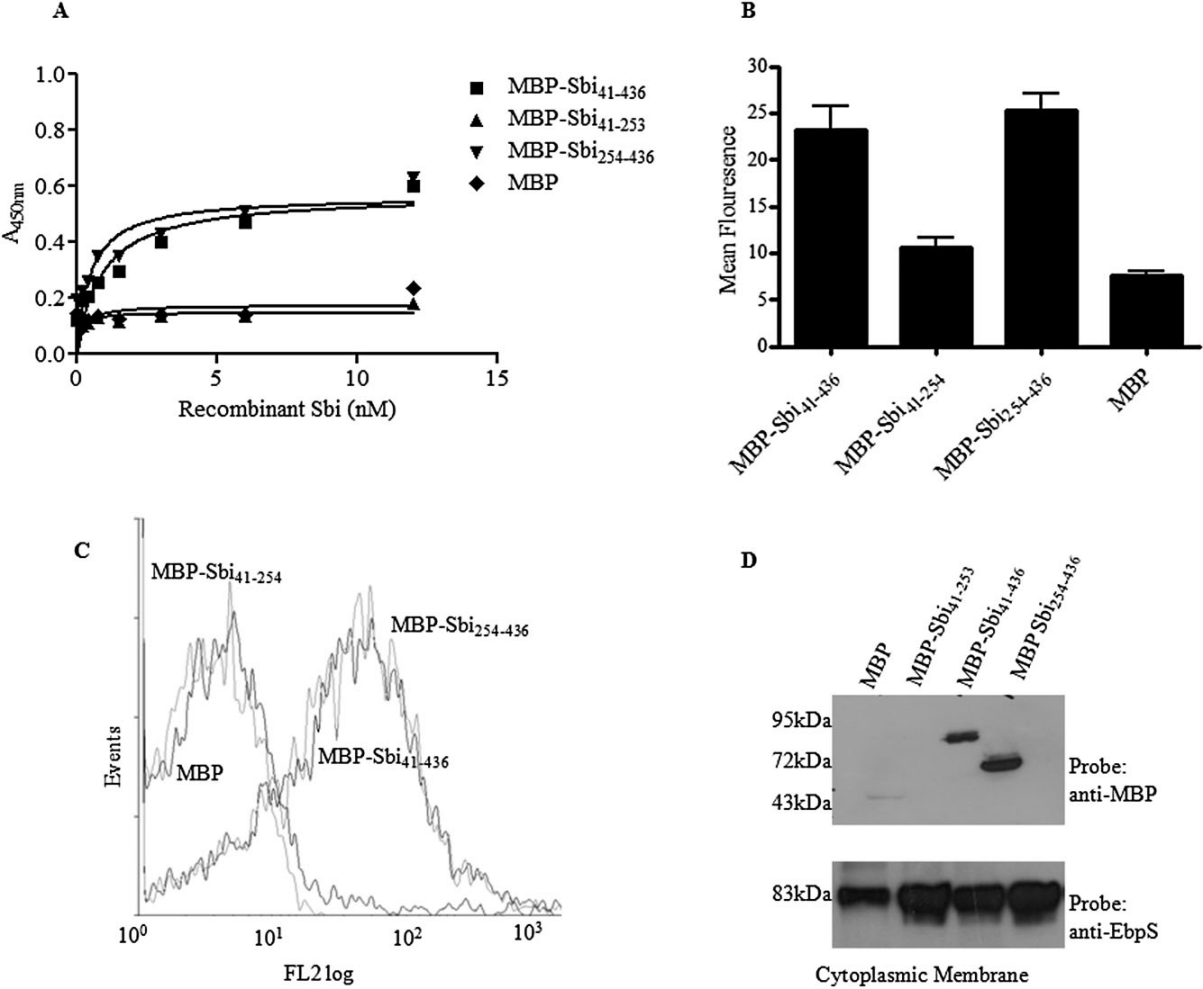
Binding of MBP–Sbi_41–436_, MBP–Sbi_41–253_ and MBP–Sbi_254–436_ to whole cells of Newman Spa^-^ Sbi^-^. A. Binding of MBP–Sbi_41–436_, MBP–Sbi_254–436_ MBP–Sbi_41–253_ and MBP to wells coated with whole Newman Spa^-^ Sbi^-^ cells. Recombinant protein binding was detected with HRP-conjugated anti-MBP IgG. Binding assay was preformed *n* = 3 times with similar results. The graph shown is a representative of one experiment with each plot in the graph representing the average of duplicate wells. B. Washed whole cells of Newman Spa^-^ Sbi^-^ were incubated with 0.5 µM MBP–Sbi_41–436_, MBP–Sbi_254–436,_ MBP–Sbi_41–253_ and MBP followed by mouse anti-MBP antiserum and FITC-labelled rabbit anti-mouse IgG. Fluorescence intensity was measured by flow cytometry. The assay was preformed *n* = 3 times. Each plot on the graph represents the average value for all three replicas. Error bars show the standard deviation. C. A representative flow cytometry trace of recombinant Sbi derivatives binding to Newman Spa^-^ Sbi^-^. D. Recombinant MBP–Sbi_41–436_, MBP–Sbi_254–436_ MBP–Sbi_41–253_ and MBP were incubated with whole cells of Newman Spa^-^ Sbi^-^ and fractionated. Cytoplasmic membrane fractions were analysed by Western blotting with HRP-conjugated anti-MBP IgG or rabbit anti-EbpS IgG followed by HRP-conjugated protein A. All immunoblotting experiments were repeated *n* = 3 times.

Recombinant MBP–Sbi binds to purified cytoplasmic membrane material with high affinity. To address whether this mode of association is similar to that of Sbi expressed by *S. aureus*, recombinant proteins MBP–Sbi_41–436_, MBP–Sbi_41–253_ and MBP–Sbi_254–436_ were separately incubated with whole cells of Newman *spa sbi*. MBP–Sbi_41–436_ and MBP–Sbi_254–436_ both bound to whole cells dose-dependently and saturably with similar half-maximal binding concentrations (0.55 ± 0.03 nM and 0.57 ± 0.02 nM respectively) (Fig. 4A). Binding of recombinant MBP–Sbi_41–436_, MBP–Sbi_41–253_, MBP–Sbi_254–436_ and MBP to whole cells of strain Newman Spa^-^ Sbi^-^ was also investigated by flow cytometry. Recombinant Sbi derivatives were separately incubated with whole cells of Newman Spa^-^ Sbi^-^ and binding was detected with monoclonal mouse anti-MBP followed by FITC-labelled rabbit anti-mouse IgG. MBP–Sbi_41–436_ and MBP–Sbi_254–436_ both bound to whole cells confirming the enzyme-linked immunosorbent assay (ELISA) result (Fig. 4B and C).

In order to determine if recombinant Sbi that bound to the bacterial cells fractionated with the cell wall or membranes during protoplast formation, and to determine if a receptor for Sbi is exposed on the cell surface, Newman Spa^-^ Sbi^-^ was incubated with MBP–Sbi_41–436_ and MBP–Sbi_254–436_, washed and then fractionated to isolate the cytoplasmic membrane. Figure 4D shows the purified cytoplasmic membrane fraction probed with HRP-conjugated anti-MBP antiserum. The ∼ 86 kDa band present in lane 3 corresponds to recombinant MBP–Sbi_41–436_ while the ∼ 63 kDa band in lane 4 corresponds to recombinant MBP–Sbi_254–436_ (Fig. 4D). These data show that a receptor for the C-terminal domain of Sbi is exposed on the cell surface and yet the added recombinant protein is associated with the membrane following fractionation of protoplasts

### Sbi binds to lipoteichoic acid

**Fig. 5:**
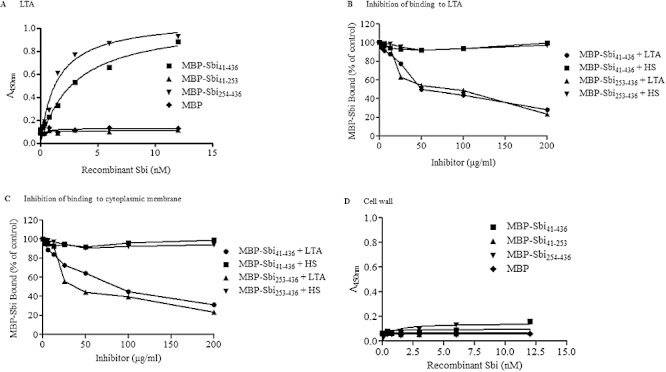
Interaction of MBP–Sbi with LTA. A. Binding of MBP–Sbi_41–436_, MBP–Sbi_254–436_ MBP–Sbi_41–253_ and MBP to LTA-coated wells. B and C. (B) Inhibition of binding of MBP–Sbi_41–436_ and MBP–Sbi_254–436_ to LTA and (C) to the purified cytoplasmic membrane fraction from *S. aureus* Newman Spa^-^ Sbi^-^. Recombinant proteins were pre-incubated with increasing concentrations of either *S. aureus* LTA or heparin sulphate (0–200 µg ml^−1^) before being added to coated microtitre plates. D. Binding of MBP–Sbi proteins to purified cell wall-coated wells. In each assay recombinant protein binding was detected with HRP-conjugated anti-MBP IgG. Each assay was preformed *n* = 3 times with similar results. The graphs shown are representatives of one experiment with each plot in the graph representing the average of triplicate wells.

The C-terminal region of Sbi has neither a sequence of hydrophobic residues sufficient to span the cytoplasmic membrane typical of integral or membrane-spanning proteins nor a lipoprotein consensus sequence that could be involved in anchoring the protein to the cytoplasmic membrane, raising the possibility that the association of Sbi with the cytoplasmic membrane could be due to binding to a membrane-associated component. Lipoteichoic acid (LTA) is an anionic polymer linked to a glycolipid anchor in the outer face of the cytoplasmic membrane and with a poly(glycerophosphate) chain that extends across the cell wall (Neuhaus and Baddiley, 2003). LTA remains associated with the protoplast after removal of the cell wall peptidoglycan by lysostaphin (Neuhaus and Baddiley, 2003). To determine if LTA could be the target for the C-terminal WrY domain of Sbi, wells of microtitre plates were coated with a constant amount of purified *S. aureus* LTA and incubated with increasing concentrations of recombinant MBP–Sbi_41–436_, MBP–Sbi_41–253_ and MBP–Sbi_254–436_. Proteins containing the C-terminal domain WrY (Sbi_41–436_ and Sbi_254–436_) were able to bind LTA in a dose-dependent and saturable manner with half maximal concentrations of 0.86 ± 0.2 nM and 0.84 ± 0.2 nM respectively (Fig. 5A). Furthermore, pre-incubation of Sbi with different concentrations of *S. aureus* LTA inhibited binding to immobilized LTA and to purified cytoplasmic membranes in a dose-dependent manner (Fig. 5B and C).

To investigate the specificity of the interaction between Sbi and LTA, MBP–Sbi was pre-incubated with different concentrations of heparin sulphate, an anionic glycosaminoglycan which consists of a repeating disaccharide unit of glucosamine and uronic acid residues and which occurs on the surface of mammalian cells (Li and Vlodavsky, 2009). Heparin sulphate (HS) was able to displace InlB from the surface of *L. monocytogenes* but was not able to inhibit the interaction of MBP–Sbi with immobilized LTA or purified cytoplasmic membrane material (Fig. 5B and C), suggesting that the interaction between LTA and Sbi is specific and not simply due to the positively charged protein binding to negatively charged residues in surface polymers.

### Can Sbi bind to purified cell wall?

The cell wall contains wall teichoic acid, a polymer of ribitol phosphate that is covalently anchored to the peptidoglycan (Neuhaus and Baddiley, 2003; Xia *et al*., 2010) as well as covalently anchored wall-associated proteins such as ClfA and Spa. The cell wall fraction of *S. aureus* Newman Spa^-^ Sbi^-^ was purified following disruption of the cells by mechanical shearing and by boiling with detergent to solubilize the cytoplasmic membrane.

The coating affinity and purity of the cell wall fraction was assessed by ELISA using (i) polyclonal anti-ClfA antibodies and (ii) polyclonal anti-EbpS antibodies, with maximum absorbencies at saturation 0.76 and 0.22 respectively. This showed that the wall fraction bound to the ELISA wells efficiently and that the level of contamination with membrane was low. MBP–Sbi_41–436_ and MBP–Sbi_254–436_ was added to the cell wall-coated ELISA wells but did not bind detectably. This shows that Sbi cannot bind to the purified cell wall fraction (Fig. 5D).

### Displacement of Sbi from purified cytoplasmic membranes by soluble LTA

**Fig. 6:**
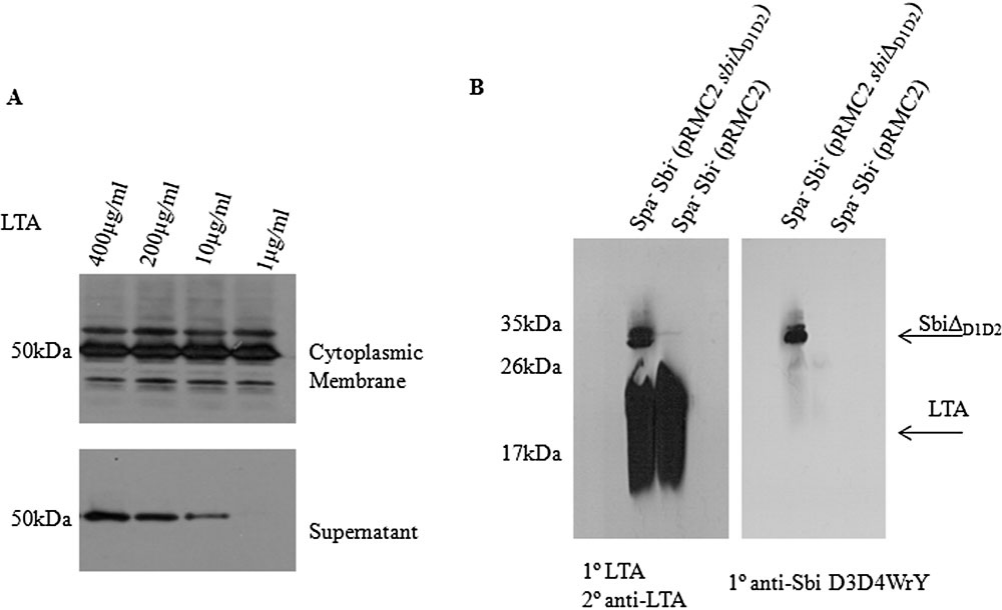
Analysis of the Sbi–LTA interaction. A. Displacement of Sbi from the cytoplasmic membrane by soluble LTA. Newman Spa^-^ cytoplasmic membrane was incubated with increasing amounts of *S. aureus* LTA (0–400 µg ml^−1^). Sbi bound to the cytoplasmic membrane or released into the supernatant was detected using rabbit anti-Sbi D3D4WrY IgG followed by HRP-conjugated goat anti-rabbit IgG. B. Far Western blotting of *S. aureus* cytoplasmic membrane fractions with LTA. Newman Spa^-^ Sbi^-^ and Newman Spa^-^ Sbi^-^ (pRMC2-*sbi*Δ_D1D2_) cytoplasmic membrane material was fractionated by SDS-PAGE, transferred to a nitrocellulose membrane incubated with purified LTA and bound LTA was detected with anti-LTA monoclonal antibody followed by HRP-linked rabbit anti-mouse IgG. All immunoblotting experiments were repeated *n* = 3 times.

In order to determine if soluble LTA could displace Sbi from the membrane of *S. aureus* cells, purified cytoplasmic membranes of the Sbi^+^ strain Newman Spa^-^ were incubated with different concentrations of LTA. Some Sbi was displaced into the supernatant in a concentration-dependent manner while the majority remained associated with the membrane (Fig. 6A). This indicates that Sbi is attached to the membrane with high affinity at least in part by binding to LTA and suggests the possibility of a second ligand.

### Detection of Sbi–LTA interaction by Far Western blotting

**Fig. 7:**
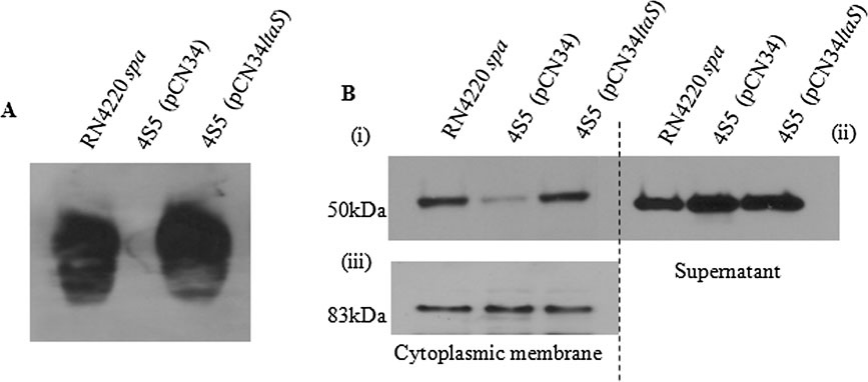
Sbi cellular location in LTA-negative strains. A. Whole-cell lysate fractions of RN4220 Spa^-^, 4S5 and 4S5 (pCN34-*ltaS*) analysed by Western immunoblotting with monoclonal mouse anti-LTA antibodies followed by HRP-conjugated rabbit anti-mouse IgG. B. Cytoplasmic membrane and culture supernatant fractions of RN4220 Spa^-^, 4S5 and 4S5 (pCN34-*ltaS*) analysed by Western immunoblotting with rabbit anti-Sbi D3D4WrY IgG and HRP-conjugated goat anti-rabbit IgG (i and ii) and rabbit anti-EbpS IgG followed by HRP-conjugated goat anti-rabbit IgG (iii). Blots shown are representative of three independent experiments. Densitometric analysis was carried out using ImageJ software. Integrated band densities were measured with correction of background. Values given are the mean ± the standard deviation of *n* = 3 experiments

The experiment described above shows that recombinant Sbi can bind to LTA. In order to determine if Sbi expressed by *S. aureus* binds LTA a Far Western blotting approach was taken. The membrane fraction of *S. aureus* Newman Spa^-^ Sbi^-^ lacking the ability to express the IgG-binding proteins Spa and Sbi from chromosomal genes but expressing SbiΔ_D1D2_ from pRMC2 was purified. The membrane fraction was separated by SDS-PAGE, transferred to a nitrocellulose membrane and probed with LTA followed by a mouse monoclonal antibody to LTA and HRP-conjugated rabbit anti-mouse IgG. The reactive band at ∼ 35 kDa corresponds to SbiΔ_D1D2_ demonstrating that Sbi expressed from *S. aureus* binds LTA (Fig. 6B). An immunoreactive band of 17–24 kDa was detected in both samples which presumably corresponds to LTA that is present in the membrane fraction (Fig. 7B). The Far Western blot was subsequently stripped, probed with anti-Sbi serum and overlaid on the original blot (Fig. 6B). A reactive band at ∼ 35 kDa was detected which corresponds to SbiΔ_D1D2_ demonstrating that it is Sbi expressed from *S. aureus* which binds LTA in the Far Western blot.

### An LTA defective mutant has decreased levels of Sbi in the cytoplasmic membrane

*Staphylococcus aureus* mutants that lack LTA can only grow under osmotically stabilizing conditions or by the acquisition of compensatory mutations (Oku *et al*., 2009; Corrigan *et al*., 2011). The RN4220 Spa^-^-derived LTA-deficient strain 4S5 contains a complete deletion of the LTA synthase gene *ltaS* and has acquired two additional mutations that permit this strain to grow and divide in the absence of LTA similar to the wild type (Corrigan *et al*., 2011). To assess the role of LTA in the localization of Sbi to the cell membrane, cytoplasmic membrane and culture supernatant fractions of wild-type RN4220 Spa^-^ and 4S5 cells grown to mid-exponential phase were isolated and analysed by SDS-PAGE and Western immunoblotting using the rabbit anti-Sbi D3D4WrY serum. An immunoreactive band of ∼ 50 kDa was detected in the membrane and supernatant fractions of both the wild-type and LTA-negative strain [Fig. 7B(i)]. However, densitometric analysis of band intensity indicated that strain 4S5 exhibited 3.4 ± 0.4-fold lower levels of Sbi in its cytoplasmic membrane but contained a 1.8 ± 0.2-fold higher level of Sbi in the supernatant fraction as compared with the wild-type strain [Fig. 7B(ii)].

To confirm the role of LTA in Sbi localization and to rule out any involvement of the suppressor mutations present in 4S5, the complementation vector pCN34-*ltaS* (Corrigan *et al*., 2011), which expresses *ltaS* from its native promoter, was electroporated into 4S5. This resulted in the restoration of LTA synthesis as judged by Western immunoblotting (Fig. 7A). Complementation with pCN34-*ltaS* also restored Sbi expression in the cytoplasmic membrane to wild-type levels (Fig. 7B). This shows that the suppressor mutations are not responsible for the reduction in Sbi in the membrane fraction and directly implicates LTA in attaching Sbi to the cell envelope. Furthermore, the level of the integral membrane protein EbpS in the cytoplasmic membrane of all strains was the same. This indicates that the inhibition of LTA expression does not affect another membrane-associated protein [Fig. 7B(iii)].

### Association of Sbi with the membrane by its C-terminal Y domain

**Fig. 8:**
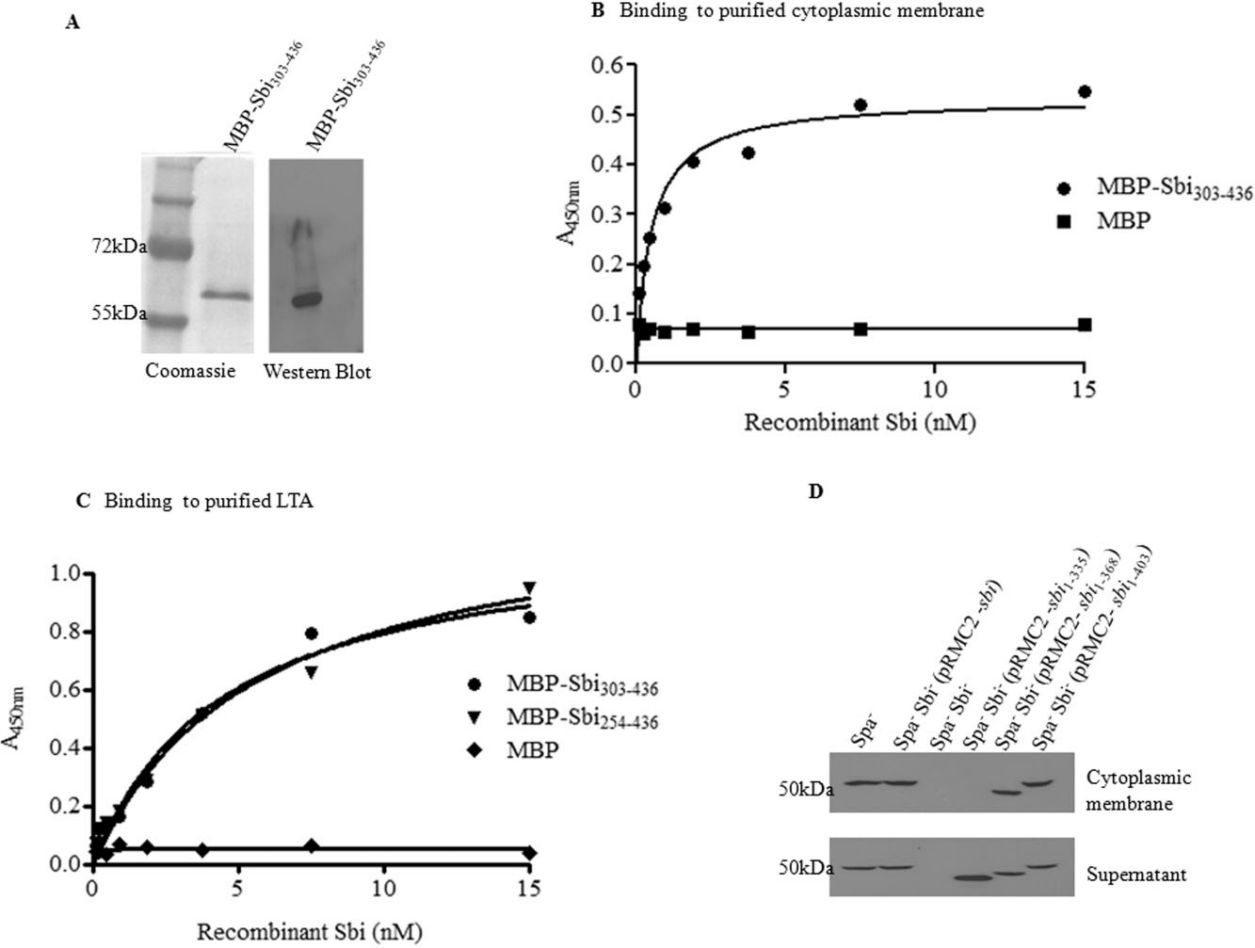
Cellular localization of Sbi C-terminal truncates. A. Coomassie stain of an SDS-PAGE gel of MBP–Sbi_303–436_. Western immunoblot of MBP–Sbi_303–436_ probed with HRP-conjugated anti-MBP IgG. B. Binding of MBP–Sbi_303–436_ and MBP to plates coated in Newman Spa^-^ Sbi^-^ cytoplasmic membrane material. C. Binding of MBP–Sbi_254–436_, MBP–Sbi_303–436_ and MBP to Newman Spa^-^ Sbi^-^ LTA-coated wells. Recombinant protein binding was detected with HRP-conjugated anti-MBP IgG. Each assay was preformed *n* = 3 times with similar results. The graphs shown are representatives of one experiment with each plot in the graph representing the average of duplicate wells. D. Cytoplasmic membrane and culture supernatant fractions of Newman Spa^-^ Sbi^-^ (pRMC2-*sbi*) C-terminal truncates analysed by Western immunoblotting with HRP-labelled rabbit IgG.

It seemed likely that the C-terminal domain Y is involved in attaching Sbi to the cytoplasmic membrane. To test this, an MBP-fusion protein was constructed that comprised the C-terminal Y domain (residues 303–436) (Fig. 3A). The protein was expressed in *E. coli* and purified by affinity chromatography. The protein's purity and integrity were verified by SDS-PAGE and Western blotting with anti-MBP serum (Fig. 8A).

Cytoplasmic membrane material purified from *S. aureus* Newman Spa^-^ Sbi^-^ was incubated in microtitre plates and coating of the surface verified with antibodies recognizing the integral membrane protein EbpS (data not shown). The membranes were incubated with MBP–Sbi_303–436_ which was able to bind in a dose-dependent and saturable manner with half maxima of 0.5 ± 0.04 nM while the MBP control was unable to bind (Fig. 8B). These results indicate that the C-terminal Y domain of Sbi binds to purified cytoplasmic membrane. MBP–Sbi_303–436_ also bound purified LTA in a dose-dependent and saturable manner with half maxima of 1 ± 0.15 nM (Fig. 8C).

To localize the residues involved, the full-length *sbi* gene and a series of deletions were cloned into the expression vector pRMC2 so that the *sbi* gene and truncates were expressed from the anhydrotetracycline-inducible promoter on the vector. The plasmids expressing full-length Sbi and the C-terminal truncates Sbi_1–335_, Sbi_1–368_ and Sbi_1–403_ were introduced into Newman Spa^-^ Sbi^-^, bacteria were grown in the presence of the inducer and cytoplasmic membrane and culture supernatant fractions analysed by SDS-PAGE and Western blotting. A 50 kDa band corresponding to Sbi was detected in the membrane and supernatant fractions of the strain expressing the wild-type Sbi. The three truncates were detected in the culture supernatant, but in the case of the shortest, Sbi_1–335_, the protein was not present in the membrane fraction and was only found in the supernatant (Fig. 8D). This suggests that the C-terminal domain Y is required to anchor the protein to the membrane and that residues 335–368 are required for efficient membrane anchoring but are not solely responsible.

### Why does Sbi occur extracellularly?

**Fig. 9:**
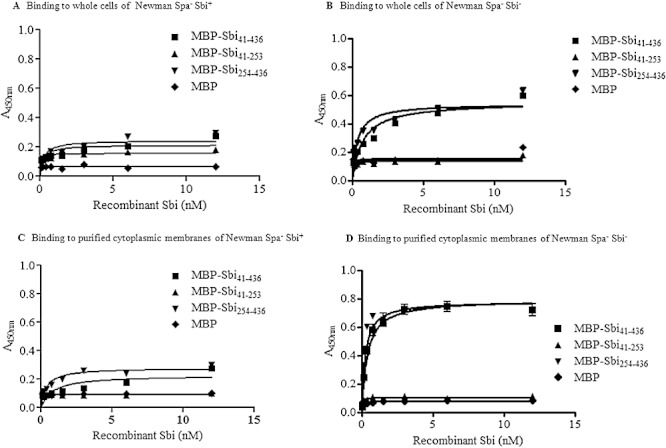
Binding of MBP–Sbi to whole cells and purified cytoplasmic membrane. Binding of MBP–Sbi_41–436,_ MBP–Sbi_254–436_ MBP–Sbi_41–253_ and MBP to wells coated with (A) *S. aureus* Newman Spa^-^ Sbi^+^ cells, (B) Newman Spa^-^ Sbi^-^ cells, (C) Newman Spa^-^ Sbi^+^ cytoplasmic membrane material and (D) Newman Spa^-^ Sbi^-^ cytoplasmic membrane material. Closed symbols and black lines refer to Newman Spa^-^. Recombinant protein binding was detected with HRP-conjugated anti-MBP IgG. Each assay was preformed *n* = 3 times with similar results. The graphs shown are representatives of one experiment with each plot in the graph representing the average of duplicate wells. Blots shown are representative of three independent experiments.

Given that recombinant MBP–Sbi protein binds to LTA exposed on the bacterial cell surface it is perhaps surprising that Sbi can occur extracellularly unless the cell-bound form saturates the surface-exposed LTA or the C-terminus of the secreted form is modified so that it cannot recognize LTA. To address this issue Newman Spa^-^ Sbi^+^ and Newman Spa^-^ Sbi^-^ cells were coated onto the surface of ELISA wells and incubated with MBP–Sbi_41–436_ and MBP–Sbi_254–436._ A reduction in absorbance of 2.3- and 3-fold occurred, respectively, when binding of MBP–Sbi to the Sbi^+^ strain was compared with the Sbi^-^ mutant (Fig. 9A and B). This suggests that a significant fraction of the LTA on the surface of Sbi^+^ cells is unavailable for binding by the secreted form of Sbi.

Similar results were obtained when purified membranes from Newman Spa^-^ Sbi^+^ and Newman Spa^-^ Sbi^-^ cells were immobilized and incubated with MBP–Sbi proteins, with a 2.4-fold and 2.7-fold reduction in binding of MBP–Sbi_41–436_ and MBP–Sbi_254–436_ respectively (Fig. 9C and D). This suggests that a significant number of sites were unavailable in the membrane material purified from the Sbi^+^ cells. To rule out the possibility that increased binding by Sbi^-^ cells and cytoplasmic membranes may be due to increased production of LTA the amount of LTA in Sbi^+^ and Sbi^-^ cells was investigated by Western blotting with anti-LTA serum. No difference in LTA expression was seen between Sbi^+^ and Sbi^-^ bacteria (data not shown).

### Can the Sbi Y domain support binding to the cytoplasmic membranes of other type I LTA expressing strains?

**Fig. 10:**
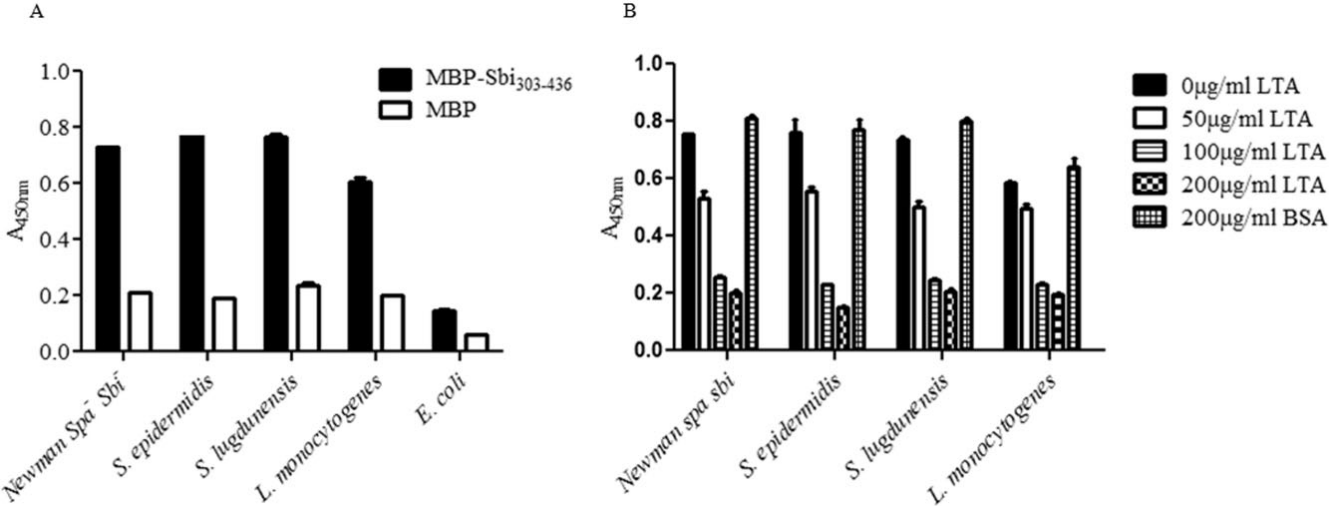
Binding of MBP–Sbi_303–436_ to the cytoplasmic membranes of Gram-positive bacteria. A. Cytoplasmic membrane material purified from *S. aureus*, *S. epidermidis*, *S. lugdunensis*, *L. monocytogenes* and *E. coli* were incubated with MBP–Sbi_303–436_ and MBP in microtitre plates. Recombinant protein binding was detected with HRP-conjugated anti-MBP IgG. B. Recombinant MBP–Sbi_303–436_ was pre-incubated with increasing concentrations of either *S. aureus* LTA or bovine serum, albumin (BSA) (0–200 µg ml^−1^) before being added to cytoplasmic membrane-coated microtitre plates. Recombinant protein binding was detected with HRP-conjugated anti-MBP IgG. Each assay was preformed *n* = 3 times with similar results. The graphs shown are representatives of one experiment with each plot in the graph representing the average of duplicate wells.

The ability of Sbi to bind to membranes (presumably via LTA) of other Gram-positive bacteria was investigated. Cytoplasmic membrane material purified from *S. aureus*, *S. epidermidis*, *S. lugdunensis*, *L. monocytogenes* and the Gram-negative bacteria *E. coli* was incubated in microtitre plates. The membranes were incubated with either MBP–Sbi_303–436_ or MBP. MBP–Sbi_303–436_ bound the cytoplasmic membranes of all five Gram-positive bacteria with a similar affinity while the MBP control was unable to bind (Fig. 10A). All five Gram-positive bacterial strains tested express type I LTA which has a poly(glycerophosphate) backbone (Neuhaus and Baddiley, 2003). In contrast MBP–Sbi_303–436_ did not bind to the cytoplasmic membrane material of *E. coli* which does not express LTA. These results indicate that the C-terminal Y domain of Sbi binds strongly to purified cytoplasmic membranes of strains expressing LTA which has a poly(glycerophosphate) backbone. Furthermore MBP–Sbi_303–436_ binding to the cytoplasmic membranes of all four Gram-positive bacteria was inhibited by pre-incubating MBP–Sbi_303–436_ with soluble purified LTA from *S. aureus* (Fig. 10B).

## Discussion

The notion that the function of the Sbi protein is to help protect *S. aureus* from innate immune defences of the host was initially based on the *in vitro* activities of the recombinant D1 and D2 domains that bind to the Fc region of IgG and the D3 and D4 domains which can bind to complement factor C3 in serum and can promote its futile consumption (Zhang *et al*., 1998; 1999; Burman *et al*., 2008). If Sbi protects cells in the same manner as protein A by binding IgG at the Fc region so that the immunoglobulin cannot act as an opsonin or promote complement fixation, the protein must be bound to the cell envelope with domains D1 and D2 exposed on the cell surface. However, if domains D3 and D4 are to promote futile consumption of C3 in the fluid phase they must do so at a distance from the cell otherwise they would actually promote opsonin formation. This requires the protein to be secreted from the cell and for the D3D4 domains of the cell-associated Sbi to be inactive.

We have recently shown that Sbi both is associated with the cell envelope (where it is at least partly displayed on the surface) and is also present extracellularly and that both forms of the protein contribute to immune evasion (Smith *et al*., 2011). Examination of the level of exposure of Sbi on the surface of whole cells indicates that the N-terminal D1 and D2 domains are available to bind IgG. In contrast only a small proportion of the D3 and D4 domains are recognized by the Fab regions of anti-D3D4WrY IgG.

The Sbi protein is attached to the cell envelope by an unusual mechanism. When cells were converted to protoplasts the cell-associated protein was not solubilized like the LPXTG-anchored protein A or ClfA. Instead it was attached to the cytoplasmic membrane fragments following lysis of protoplasts and sedimentation. Purified recombinant MBP–Sbi bound to immobilized membrane fragments prepared from an Sbi^-^ mutant. Finding that recombinant Sbi bound to purified lipoteichoic acid both dose-dependently and saturably suggests that LTA is the ligand in the cell envelope bound by Sbi when it is secreted across the membrane of growing cells. Also, reaching saturation in the binding assays is important because it is indicative of a specific interaction. Furthermore MBP–Sbi could not bind to purified cell wall material.

**Fig. 11:**
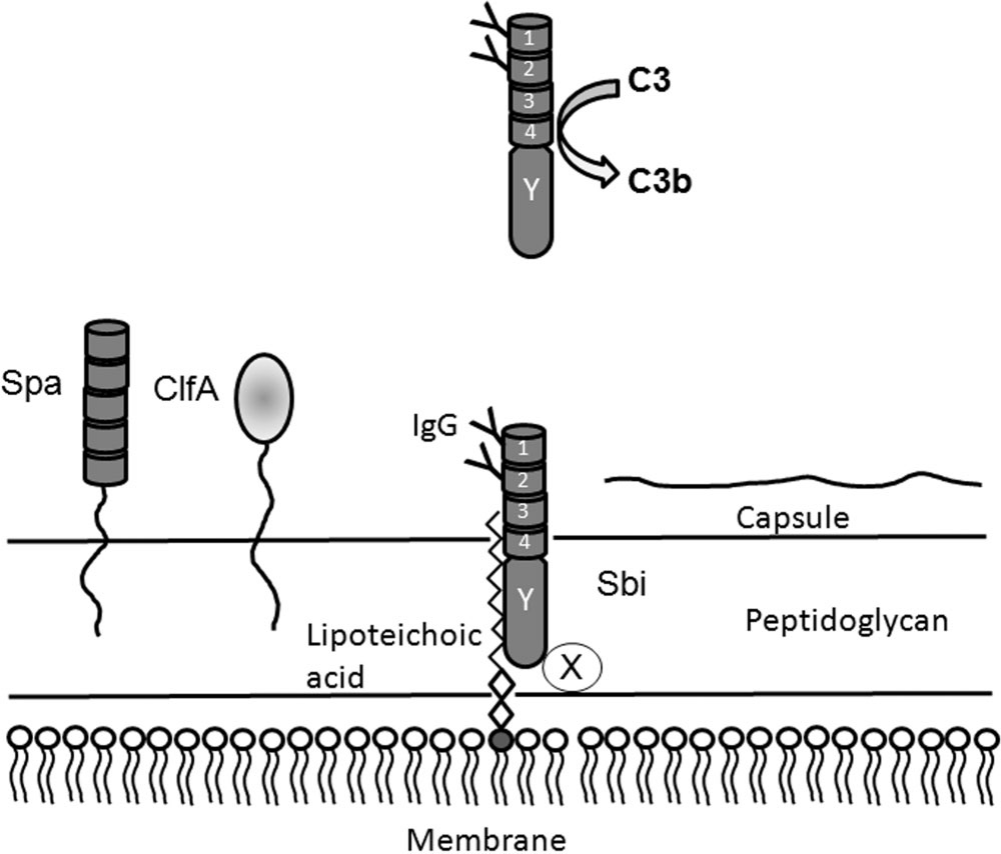
Proposed model for Sbi cellular localization and surface expression. The diagram shows Sbi binding to LTA and to a putative second membrane component (X). Only one face of the lipid bilayer is shown. For LTA the jagged line represent polyglycerol phosphate repeats and the diamond the disaccharide linker. The IgG-binding domains D1D2 are biologically active when Sbi is associated with the cell membrane. Whereas D3D4 are biologically active only when secreted. The wall-anchored proteins Spa and ClfA are shown for context.

In support of our contention that LTA is a ligand for attaching Sbi to the cell envelope is (i) the specific binding of rSbi both to purified LTA and to the membrane fraction, (ii) inhibition of binding of rSbi to the membrane fraction by soluble LTA, (iii) partial displacement of Sbi from the membrane fraction by soluble LTA, (iv) binding of rSbi to the surface of whole cells where LTA is known to be exposed, (v) a reduction in the level of Sbi in the cytoplasmic membrane of an LTA-negative mutant, and (vi) the ability to bind to the membranes of other Gram-positive bacteria with type I LTA in their cytoplasmic membranes. However, LTA might not be the only ligand for Sbi in the membrane. High concentrations of soluble LTA could only partially displace bound Sbi. Figure 11 summarizes our current understanding of the association of Sbi with the cell envelope and its role in immune evasion. It shows membrane-associated Sbi partially exposed on the cell surface with the N-terminal D1D2 domains able to bind IgG. Extracellular Sbi cannot attach to the cell because most of the LTA is already occupied ensuring that at least some Sbi is available and possibly triggers C3 metabolism as described by Burman *et al*. (2008). The secreted form of Sbi can also bind IgG but this does not provide any protection from opsonophagocytosis (Smith *et al*., 2011). The behaviour of Sbi in cell fractionation experiments and its ability to bind to LTA resembles internalin B (InlB) of *L. monocytogenes* (Jonquieres *et al*., 1999). InlB binds to the human growth hormone receptor Met on mammalian cells and triggers bacterial internalization by receptor-mediated endocytosis (Mengaud *et al*., 1996; Braun *et al*., 1998; Shen *et al*., 2000). In order to act as an invasin InlB must be able to promote clustering of the Met receptor in the host cell membrane and trigger the signalling that leads to cytoskeletal rearrangements and endocytosis (Niemann, 2011). The ability of a secreted protein to perform this seems paradoxical until one considers the role of heparin sulphate proteoglycans that are bound on the surface of mammalian cells (Marino *et al*., 2002). HSP is able to displace cell-associated InlB and to promote its release from the cell surface. It does so by binding to the GW repeats that contain the binding sites for LTA. It is likely that HSP–InlB complexes cooperate to trigger Met receptor clustering.

A major difference between the association of InlB and Sbi with LTA is that the latter does not bind to HSPs *in vitro* and cannot be displaced easily from the membrane. This is likely to be due to the highly specific interaction that occurs between Sbi and LTA whereas the binding of InlB to LTA appears to be much weaker and is probably non-specific and based on binding of GW repeats to highly negatively charged polymers.

In conclusion Sbi is an immune evasion protein that helps *S. aureus* to evade neutrophil-mediated phagocytosis in human blood. It is present both in the medium and in the cell envelope where it is displayed on the surface of the bacterium. Both the secreted and cell-bound forms of Sbi are required for full protection. The association of Sbi with the cell envelope involves a specific interaction with LTA and possibly another component of the membrane.

## Experimental procedures

### Bacterial strains, plasmids and growth conditions

Bacterial strains and plasmids used in this study are listed in Table S1. *S. aureus* was routinely grown on tryptic soy agar (TSA) or broth (TSB) at 37°C with shaking. *E. coli* strains were grown on L agar (Difco) or in L broth at 37°C with shaking. Antibiotics (Sigma) were added as required: chloramphenicol (Cm) at 10 µg ml^−1^, erythromycin (Em) at 10 µg ml^−1^, tetracycline (Tc) at 2 µg ml^−1^, kanamycin (Ka) at 50 µg ml^−1^ and ampicillin (Ap) at 100 µg ml^−1^.

### Isolation of an *sbi* mutant by allelic replacement

To inactivate the *sbi* gene, DNA fragments comprising 900 bp upstream and 740 bp downstream of *sbi* were amplified by PCR from genomic DNA and cloned together with an *ermC* cassette (from pTS*ermC*) between the HindIII and BamHI sites of plasmid pBluescript. The construct was then ligated to pTS*tet*K, a plasmid that is temperature sensitive for replication in *S. aureus*, and the resulting chimeric plasmid was designated pES2. pES2 was electroporated into *S. aureus* strain RN4220 then transferred into *S. aureus* Newman to achieve integration of the *ermC* gene into the genome by homologous recombination by selecting on agar containing Em at 43°C. After several cycles of growth in broth at 28°C and at 43°C colonies were selected on Em agar and tested for loss of Tc^r^. The *sbi* mutation was validated by PCR and Western immunoblotting.

### Cell fractionation

Solubilized cell wall proteins were obtained as follows. Bacterial cultures of *S. aureus*, *S. epidermidis* and *S. lugdunensis* were harvested by centrifugation at 2000 *g* for 10 min at 4°C, washed in phosphate-buffered saline (PBS) and resuspended in 1/20 volume of protoplast buffer [50 mM Tris-HCl, 20 mM MgCl_2_, 30% (w/v) raffinose, pH 7.5] containing complete mini EDTA-free protease inhibitors (Roche). Cell wall proteins were solubilized by digestion with lysostaphin (200 µg ml^−1^) at 37°C for 15 min. Protoplasts were harvested by centrifugation at 6000 *g* for 15 min and the supernatant was retained as the cell wall fraction. Protoplasts were sedimented by centrifugation at 6000 *g* and resuspended in protoplast buffer with protease inhibitors. Protoplast pellets were washed once and resuspended in ice-cold 50 mM Tris-HCl, pH 7.5 containing protease inhibitors and DNase (80 µg ml^−1^). Protoplasts were lysed on ice by vortexing. The membrane fraction was obtained by centrifugation at 40 000 *g* for 1 h at 4°C. The supernatant was retained as the cytoplasm fraction. The pellet was washed once with ice-cold lysis buffer and finally resuspended in 50 mM Tris-HCl, pH 7.5. The culture supernatant was filtered through a 0.45 µm filter and proteins were precipitated by addition of a 1:20 volume of ice-cold 100% w/v trichloroacetic acid (TCA).

*Listeria monocytogenes* cytoplasmic membranes were isolated by the same method as described above with the following additions. Bacterial cultures were harvested by centrifugation at 2000 *g* for 10 min and cells were washed twice in PBS. An OD_600_ of 10 was resuspended in 250 µl of digestion buffer (20 mM Tris-HCl, 10 mM MgCl_2_, 500 mM sucrose, pH 7.5). Complete EDTA-free protease inhibitor cocktail (70 µl of a 10× stock), mutanolysin (1000 U ml^−1^) and lysozyme (1 mg ml^−1^) were added to the cells and incubated at 37°C for 20 min. Protoplasts were harvested by centrifugation at 3500 *g* for 15 min. Cell envelope material from *E. coli* was isolated as follows. Cultures were harvested by centrifugation at 2000 *g* for 10 min and cells were washed twice in PBS. An OD_600_ of 10 was resuspended in 10 ml of PBS containing protease inhibitors (Roche), lysozyme (200 µg ml^−1^) and DNase (3 µg ml^−1^) and allowed to stand on ice for 1 h. Cells were lysed by repeated passage through a French Pressure Cell. The lysate was centrifuged at 20 000 *g* for 15 min at 4°C in a Sorvall SS-34 rotor and the pellet was retained as the cell envelope fraction. The pellet was washed once with ice-cold lysis buffer and the pellet containing the cell envelope fraction was finally resuspended in 1 ml of ice-cold lysis buffer.

### SDS-PAGE

Protein samples were diluted in final sample buffer [125 mM Tris-HCl, pH 6.8, 4% (w/v) SDS, 20% (v/v) glycerol, 10% (v/v) β-mercaptoethanol and 0.002% (w/v) bromophenol blue] and boiled for 5 min. Samples were loaded onto acrylamide gels (3% stacking and 12% separating gel) and separated by electrophoresis (Laemmli, 1970) at 120 V after which proteins were visualized by Coomassie blue staining or electroblotted onto PVDF membranes (Roche) for Western immunoblotting.

### Western immunoblotting

Proteins were electroblotted onto PVDF membranes (Roche) for 1 h at 100 V using a wet transfer cell (Bio-Rad). Membranes were incubated for 1 h at 4°C in TS buffer (10 mM Tris-HCl, pH 7.4, 150 mM NaCl) containing 10% (w/v) skimmed milk (Marvel) (Marvel TS). Next, horseradish peroxidise-conjugated antibodies or primary antibodies diluted in Marvel TS were incubated with the membranes for 1 h at room temperature with shaking. Unbound antibody was removed by three 10 min washes with TS buffer containing 0.01% Tween. Where necessary secondary antibodies (HRP-conjugated) diluted in Marvel TS were then incubated with the membranes for 1 h at room temperature with shaking. Unbound secondary antibody was removed by washing three times with 0.05% Tween/TS buffer and developed with chemiluminescent substrate LumiGlo (New England Biolabs). Blots were exposed to X-Omat autoradiographic film (Kodak).

### Whole-cell immunoblots

Cells were washed twice in PBS and adjusted to an OD_600_ of 1. Doubling dilutions (5 µl) were dotted onto a nitrocellulose membrane (Protran). The membrane was blocked for 1 h with Marvel TS. Specific anti-Sbi D3D4WrY serum was diluted in Marvel TS buffer and incubated with the membrane for 1 h at room temperature with shaking and washed three times with TS buffer to remove unbound antibody. The secondary antibody was HRP-labelled goat anti-rabbit IgG. The membrane was developed in the dark using the chemiluminescent substrate LumiGlo (New England BioLabs)

### Anti-Sbi serum

Antibodies were raised in specific pathogen free rabbits to recombinant Sbi_153–436_ and the immunoglobulin fraction was purified. Antibodies to recombinant Sbi_153–436_ were affinity-purified to remove antibodies that cross-reacted with other *S. aureus* proteins.

### Construction of Sbi C-terminal truncates

pRMC2 plasmids that expressed N-terminal and C-terminal truncates of Sbi were generated by PCR. Amplimers were cloned between the KpnI and BglII sites in pRMC2 to create pRMC2-*sbi*_1–335_, pRMC2-*sbi*_1–368_, pRMC2-*sbi*_1–403_ and pRMC2-*sbi*ΔD1D2.

### Expression and purification of recombinant MBP-tagged Sbi

For expression of recombinant Sbi, pMAL-c2G constructs were purified from *E. coli* XL1-Blue and transformed into *E. coli* TB1 cells. Overnight cultures (20 ml) were inoculated into fresh medium (1:50) and grown to an OD_600_ of 0.5. IPTG was added to a concentration of 1.5 mM and the culture was grown for a further 3 h. Cells were harvested by centrifugation at 7000 *g* for 10 min at 4°C. The pellet was resuspended in PBS containing protease inhibitor (Roche), lysozyme (200 µg ml^−1^) and DNase I (3 µg ml^−1^) and allowed to stand on ice for 1 h. Cells were lysed by repeated passage through a French Pressure Cell. Cell debris was removed by centrifugation at 7000 *g* for 30 min at 4°C and the supernatant filtered through a 0.45 µm filter. The MBP-fusion proteins were purified using an amylose column (Bio-Rad). Proteins were eluted using 10 mM maltose in amylose column buffer and samples were analysed by SDS-PAGE for presence of the recombinant protein. Positive fractions were pooled and dialysed against PBS. Recombinant MBP-fusion proteins had approximate M_w_s ranging from 86 kDa (Sbi_41–436_) to 66 kDa (Sbi_41–253_ and Sbi_254–436_). Protein concentrations were determined using the BCA protein assay kit (Pierce) according to the manufacturer's protocol.

### Enzyme-linked immunosorbent assay (ELISA)

Nunc Maxisorp Immunoplates were coated with OD_600_ 10 of purified cytoplasmic membrane or whole cells of *S. aureus* overnight at 4°C in 50 mM sodium carbonate buffer pH 9.6. Coating was verified with anti-EbpS and anti-ClfA antibodies respectively. Wells were blocked with 5% BSA in PBS for 2 h at 37°C. Between each, wells were washed 3× with PBS. Increasing concentrations (0–12 µM) of recombinant Sbi_41–436_, Sbi_41–253_ and Sbi_254–436_ proteins were added to coated wells for 2 h at 37°C and detected with HRP-conjugated anti-MBP IgG (Bio Labs) and 3,3′, 5,5′-tetramethylbenzidine (TMB), the reaction was stopped with 2 M H_2_SO_4_ and the results read at 450 nm.

Plates were coated with 5 µM LTA (Sigma) overnight at 4°C in 50 mM sodium carbonate buffer pH 9.6. Coating A was verified with an LTA-specific monoclonal antibody. Wells were blocked with 5% BSA in PBS for 2 h at 37°C. Between each step wells were washed 3× with PBS. Increasing concentrations (0–12 nM) of recombinant Sbi_41–436_, Sbi_41–253_ and Sbi_254–436_ proteins were added to coated wells for 2 h at 37°C and detected as described above. Half maximum binding concentrations were calculated using GraphPad Prism version 4.00 for Windows, GraphPad Software, San Diego, California, USA. ELISA-type binding graphs shown throughout this article are graphs of individual experiments that are representative of three independent experiments. Each plot represents the average of duplicate or triplicate wells. Half maxima values represent the mean of three independent experiments ± standard deviation.

### Cell wall preparation

Cells from a stationary-phase culture of strain Newman Spa^-^ Sbi^-^ were adjusted to an OD_600_ 100 in PBS and were resuspended in 1.5 ml of lysis buffer containing protease inhibitors, DNase and RNase (80 µg ml^−1^). The cell suspension was transferred to a blue cap FastRNA tube and shaken in a Fastprep™ cell disrupter at speed 6 for 40 s. This was repeated 12 times with cooling on ice for 1 min between cycles. Cell lysis was monitored by phase-contrast microscopy. Lysates were then centrifuged for 2 min at 3000 *g* to pellet any remaining whole cells. The supernatant containing the cell wall fragments was boiled in 4% SDS for 2 h to remove cytoplasmic and membrane contaminates and then washed in deionized water to remove SDS. The resulting lysates were centrifuged for 15 min at 15 000 *g* to sediment the cell wall fragments.

### Inhibition of Sbi binding to purified cytoplasmic membrane and immobilized LTA with LTA

Nunc Maxisorp Immunoplates were coated with OD_600_ 10 of purified cytoplasmic membrane or LTA (5 µM) overnight at 4°C in 50 mM sodium carbonate buffer pH 9.6. Wells were blocked with 5% BSA in PBS for 2 h at 37°C. Between each step wells were washed 3× with PBS. Recombinant MBP Sbi_41–436_, Sbi_41–253_ and Sbi_254–436_ proteins were incubated with increasing concentrations of LTA or heparin sulphate (0–200 µg ml^−1^) for 1 h at 37°C before being added to coated wells for 2 h at 37°C. MBP protein binding was detected as described above.

### Fractionation of exogenously added recombinant Sbi with the cytoplasmic membrane

Newman Spa^-^ Sbi^-^ cells (OD_600_ = 5) were washed twice in PBS and incubated in PBS with 5 µg ml^−1^ of each recombinant protein Sbi_41–436_, Sbi_41–253_ and Sbi_254–436_ for 1 h at 37°C. Bacteria were pelleted by centrifugation for 5 min at 14 000 *g*. Cell fractionation was repeated as described above. Equivalent amounts of material separated by 10% SDS-PAGE and probed with HRP-conjugated anti-MBP antibodies and detected as described above.

### Displacement of Sbi from the cytoplasmic membrane with LTA

Cytoplasmic membrane fractions of Newman *spa* were prepared as described above and a sample (OD_600_ 1) was incubated in PBS with various concentrations of *S. aureus* LTA (0–400 µg ml^−1^) for 1 h at 37°C and then pelleted by centrifugation 40 000 *g* for 1 h at 4°C. The pellets were resuspended in 50 mM Tris-HCl, pH 7.5. The supernatants were filtered through a 0.45 µm filter and proteins were concentrated by addition of a 1:20 volume of ice-cold 100 w/v TCA.

### Far Western blotting of *S. aureus* cytoplasmic membrane fractions with LTA

Cytoplasmic membrane fractions of Newman *spa* were prepared as described above. Proteins were electroblotted onto PVDF membranes (Roche) for 1 h at 100 V using a wet transfer cell (Bio-Rad). Membranes were blocked with Marvel TS and incubated with 2.5 µg ml^−1^ LTA in TS/Marvel for 1 h at room temperature. Between each subsequent step membranes were washed 3× in TS/with Tween 0.05%. Bound LTA was detected with an LTA (polyglycerolphosphate)-specific monoclonal antibody (clone 55, HyCult Biotechnology) followed by HRP-linked anti-mouse IgG (Cell Signalling).

### Purification of lipoteichoic acid from *S. aureus*

Lipoteichoic acid was isolated from *S. aureus* strain RN4220 as described previously (Grundling and Schneewind, 2007). Purity was confirmed by NMR analyses.

### Densitometric analysis

Densitometric analysis was carried out using ImageJ software from the National Institute of Health (NIH). Integrated band densities were measured with correction for background. Values shown are the means of three independent experiments ± the standard deviation.
