# Supplementary material for: The immune evasion protein Sbi of Staphylococcus aureus occurs both extracellularly and anchored to the cell envelope by binding lipoteichoic acid
Source: Mol Microbiol. 2012 Jan 18;83(4):789–804. doi: 10.1111/j.1365-2958.2011.07966.x (PMC3378709; doi:10.1111/j.1365-2958.2011.07966.x)
Supplement: Supplementary file 1 [file mmi0083-0789-SD1.pdf]

Supplementary Table S1. Strains and plasmids

**Strains/Plasmids**	**Relevant Characteristics**	**References**
RN4220	Restriction-deficient derivative of 8325-4	(Kreiswirth *et al.*, 1983)
Newman	NCTC 8178.	(Duthie & Lorenz, 1952)
Newman Spa$^-$	Newman *spa*::Ka^r	(Higgins *et al.*, 2006)
Newman Sbi$^-$	Newman *sbi*::Em^r	This study
Newman Spa$^-$ Sbi$^-$	Transduction of *sbi*::Em^r into Newman *spa*::Ka^r	This study
RN4220 Spa$^-$	Spa$^-$ derivative of RN4220	(Grundling & Schneewind, 2007)
4S5	LTA-defective mutant of RN4220 *spa* with suppressor mutation in *gdpP*.	(Corrigan *et al.*, 2011)
S. epidermidis	RP62A	(Gill *et al.*, 2005)
L. monocytogenes	EGD serotype 1/2a	(Mackaness, 1962)
S. lugdunensis	N920143	(Heilbronner *et al.*, 2011)
pCN34	*S. aureus-E. coli* shuttle vector. Ka^r	(Charpentier *et al.*, 2004)
pCN34-*ltaS*	pCN34 encoding *ltaS* from its natural promoter. Ka^r	(Corrigan *et al.*, 2011)
pMAL-c2G	Expression vector for N-terminal maltose binding protein (MBP) fusion proteins	New England Biolabs
pMAL-c2G-$sbi_{41\text{-}436}$	pMAL-c2G encoding $Sbi_{41\text{-}436}$.Ap^r	This study
pMAL-c2G-$sbi_{41\text{-}253}$	pMAL-c2G encoding $Sbi_{41\text{-}253}$.Ap^r	This study
pMAL-c2G-$sbi_{254\text{-}436}$	pMAL-c2G encoding $Sbi_{254\text{-}436}$.Ap^r	This study
pMAL-c2G-$sbi_{303\text{-}436}$	pMAL-c2G encoding $Sbi_{303\text{-}436}$.Ap^r	This study
pRMC2	Derivative of pALC2073. Anhydrotetracycline-inducible expression vector for *S. aureus*.	(Corrigan & Foster, 2009)
pRMC2-*sbi*	pRMC2 derivative encoding full length Sbi.	This study

pRMC2-*sbi*$_{1\text{-}335}$	pRMC2 derivative encoding Sbi$_{1\text{-}335}$.	This study
pRMC2-*sbi*$_{1\text{-}368}$	pRMC2 derivative encoding Sbi$_{1\text{-}368}$.	This study
pRMC2-*sbi*$_{1\text{-}403}$	pRMC2 derivative encoding Sbi$_{1\text{-}403}$.	This study
pRMC2-*sbi*Δ_{D1D2}	pRMC2 derivative encoding SbiΔ_{D1D2}.	(Smith *et al.*, 2011)

Charpentier, E., Anton, A.I., Barry, P., Alfonso, B., Fang, Y. and & Novick, R.P. (2004) Novel cassette-based shuttle vector system for gram-positive bacteria. *Appl Environ Microbiol* **70**: 6076-6085.

Corrigan, R. M., Abbott, J.C., Burhenne, H., Kaever, V. and Grundling, A. (2011) c-di-AMP is a new second messenger in *Staphylococcus aureus* with a role in controlling cell size and envelope stress. *PLoS Pathog* **7**: e1002217.

Corrigan, R. M. and Foster, T.J. (2009) An improved tetracycline-inducible expression vector for *Staphylococcus aureus*. *Plasmid* **61**: 126-129.

Duthie, E. S. and Lorenz, L.L. (1952) Staphylococcal coagulase; mode of action and antigenicity. *J Gen Microbiol* **6**: 95-107.

Gill, S. R., Fouts, D.E., Archer, G.L, Mongodin, E.F., Deboy, R.T., Ravel, J., *et al*. (2005) Insights on evolution of virulence and resistance from the complete genome analysis of an early methicillin-resistant *Staphylococcus aureus* strain and a biofilm-producing methicillin-resistant *Staphylococcus epidermidis* strain. *J Bacteriol* **187**: 2426-2438.

Grundling, A. and Schneewind, O. (2007) Genes required for glycolipid synthesis and lipoteichoic acid anchoring in *Staphylococcus aureus. J Bacteriol* **189**: 2521-2530.

Heilbronner, S., Holden, M.T., van Tonder, A., Geoghegan, J.A., Foster, T.J., Parkhill, J. and Bentley, S.D. (2011) Genome sequence of *Staphylococcus lugdunensis* N920143 allows identification of putative colonization and virulence factors. *FEMS Microbiol Lett* **322**: 60-67.

Higgins, J., Loughman, A., van Kessel, K.P., van Strijp, J.A and & Foster, T.J. (2006) Clumping factor A of *Staphylococcus aureus* inhibits phagocytosis by human polymorphonuclear leucocytes. *FEMS Microbiol Lett* **258**: 290-296.

Kreiswirth, B. N., Lofdahl, S., Betley, M.J., O'Reilly, M., Schlievert, P.M., Bergdoll M.S and Novick, R.P. (1983) The toxic shock syndrome exotoxin structural gene is not detectably transmitted by a prophage. *Nature* **305**: 709-712.

Mackaness, G. B. (1962) Cellular resistance to infection. *J Exp Med* **116**: 381-406.

Smith, E. J.,Visai, L., Kerrigan, S.W., Speziale, P. and Foster, T.J. (2011) The Sbi protein is a multifunctional immune evasion factor of *Staphylococcus aureus*. *Infect Immun* **79**: 3801-3809.
